# COVID-19 and School Activities in Italy

**DOI:** 10.3390/v12111339

**Published:** 2020-11-23

**Authors:** Giovanni Sebastiani, Giorgio Palù

**Affiliations:** 1 Istituto per le Applicazioni del Calcolo “Mauro Picone”, Consiglio Nazionale delle Ricerche, 00185 Rome, Italy; 2 Dipartimento di Matematica “Guido Castelnuovo”, Sapienza University, 00185 Rome, Italy; 3 Department of Mathematics and Statistics, UIT The Arctic University of Norway, N-9037 Tromsø, Norway; 4 Department of Molecular Medicine, University of Padua, 35121 Padua, Italy; giorgio.palu@unipd.it

After a linear growth during September, the diffusion in Italy of SARS-CoV-2, responsible for COVID-19, has been growing exponentially since the end of that month with a doubling time approximately equal to one week. This has had an impact on public health, with the numbers of both ordinary and intensive care beds growing exponentially with the same doubling time. Different factors could have contributed to this phenomenon. Among them, school activity is expected to have played a major role, as indicated by published works dealing with the first phase of the pandemic after lockdown [1,2]. However, other measures were introduced that could have been responsible for the effect. Here, an exponential increase in the percentage frequency of SARS-CoV-2-positive cases began approximately two weeks after schools restarted in September 14, the same length of the time interval between the start of the national lockdown in March 12 and the location of the following incidence peak in March 24. Furthermore, there were no other known factors present, as, for example, work activities started at the beginning of September. Therefore, we strongly believe that the school restart has been the major factor in COVID-19 exponential increase, especially in connection to public transportation used by students. At the moment, strong measures of different types are being introduced in Italy and in other countries to limit the diffusion of the pandemic. Therefore, this work could be of great help to find the best measures to adopt. 

The influence of school attendance on the COVID-19 pandemic spread in the US has recently been addressed [1,2]. A significant direct association between decreased incidence and mortality rate of COVID-19 and statewide school closure of all grades (in all 50 states) was reported. However, other confounding factors could have played a role, such as physical distancing measures, non-essential business closures, contacts with parents and care-givers and optimal timing for intervention in different environments. All of this could impair our ability to predict how measures such as school attendance and closure would facilitate the pandemic spread or its containment, respectively. In a very recent paper using data from Southampton, UK [3], a significant increase in rhinovirus circulation was observed around 2 weeks after the reopening of schools in September 2020, and a similar occurrence was assumed for SARS-CoV-2.

Here, we report the influence of school closure and reopening on COVID-19 incidence in Italy, with the intent of evaluating such a phenomenon in a territorial context different from the US, in isolation from other interfering factors. Sampling was executed to identify subjects with SARS-CoV-2 by trained personnel operating at hospital first-aids or at specially prepared locales for diagnosis and screening, set up at schools and at centers for prevention and public health. Operational procedures for oropharyngeal and nasal swabs were executed according to the Italian guidelines licensed by Istituto Superiore di Sanità (ISS). Nylon fiber flocked swabs were used for sample collection (Copan Flock Technologies S.r.l. Brescia). Swabs contained an exclusive shaft fraction point for rapid material transfer to an appropriate vial containing a universal transport medium. Specimens were immediately processed at local microbiology and virology laboratories by means of multiplex realtime RT-PCR. Basically, two technologies were used for SARS-CoV-2 gene target amplification, Seegene and Roche, whose diagnostic systems were both endowed with a CE-IVD certificate (European validation procedure for in vitro diagnostics) and validated by ISS. 

Italy adopted a generalized lockdown on March 12 and gradually resumed all activities from May 4 to June 15, the day when borders were reopened. Only schools of all levels, from kindergarten to universities, were kept closed for physical attendance, while teaching was pursued by telematic means. Schools reopened on September 14 and 24 for 8 million students. After lockdown started, the curve of COVID-19 incidence began to drop from 7000 to a few hundred cases per day, with the lowest level reached in the mid of July (180 cases), a decline mirroring that of other European nations [4]. COVID-19 incidence started to slightly increase at the beginning of August, but the curve really became exponential at the end of September (see Figure 1). The time lag between school reopening and the start of the exponential phase was approximately two weeks, as also found in [3], close to the length of the time interval between the start of the Italian national lockdown and the location of the incidence peak (see Figure 2). We recall that two weeks is the average time between the infection of a subject and its registration. The schools reopening was the only new event occurring in Italy within this time-frame. Work activities began at the beginning of September, i.e., two weeks before schools started, with same smart working percentage as before. Differently from previous years, very few Italian or foreigner tourists were moving within Italy during September. Safety distance, assembly bans, mask use and all other precautions were maintained before and after schools reopening. The climate was extraordinary mild in September, a condition which directly or indirectly could have not influenced virus transmissibility. In addition, rhinovirus and flu virus circulation was almost absent in September.

More than 75% of new cases were coming in that period from familial outbreaks [5], an element that would point towards a new causative event in SARS-CoV-2 transmission, such as the resumed activities and movements of a few million students. In addition, a high percentage of asymptomatic subjects, especially concentrated at a young age, was estimated by a serological study performed at the end of the lockdown in Italy [6], and a low mean age of positive subjects in the period of school reopening was found [5]. Furthermore, rigorous measures were implemented in schools to limit virus spread, while, due to insufficient transportation, students were often not able to a maintain suitable distance from each other. Moreover, for social reasons, students were meeting outside schools far more often than before, frequently not wearing a face mask. For all the above facts, one can assume that students were spreading the virus among themselves, mainly outside schools, with most of them being asymptomatic. Then, at home, students infected other members of their family, with those older more likely to be symptomatic. This progressively increased the pressure on hospitals.

In order to make schools run safely in an ordinary way and not by remote learning, the problem of transportation should be solved and school times should be very different from working times, e.g., in the afternoon for high schools. In addition, serious checking to ensure that young people wear face masks outside school should be made. Moreover, contact tracing should be better implemented for students and their relatives, significantly increasing the use of the app ‘immuni’. Finally, intensive fast and/or pool-testing of students and school workers should be performed regularly, based on statistical criteria. Although there is still uncertainty as to whether children and youth can efficiently be infected and transmit the virus [7], our results confirm the Auger report that schools and associated activities could affect the COVID-19 spread. In addition, our report gives insights into contentious issues, such as the right to education and public health consequences.

## Figures and Tables

**Figure 1:**
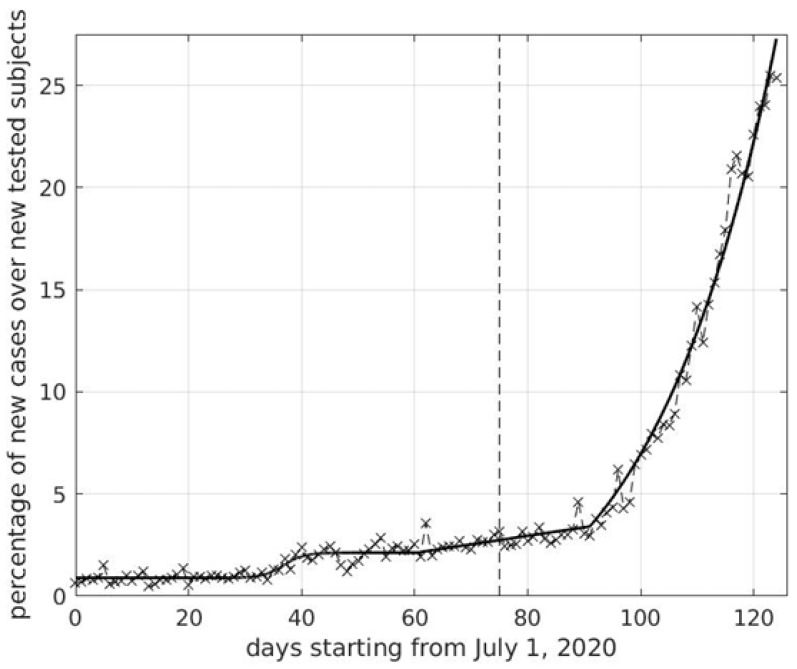
Percentage of SARS-Cov-2 positive cases over tested subjects in Italy versus number of days starting from July 1. The dashed vertical segment corresponds to schools re-opening on September 14. At about 14 days after school re-opening, linear growth is replaced by exponential. Data are made publicly available by Italian Civil Protection at https://github.com/pcm-dpc/COVID-19/tree/master/dati-regioni (accessed on 2 November 2020).

**Figure 2:**
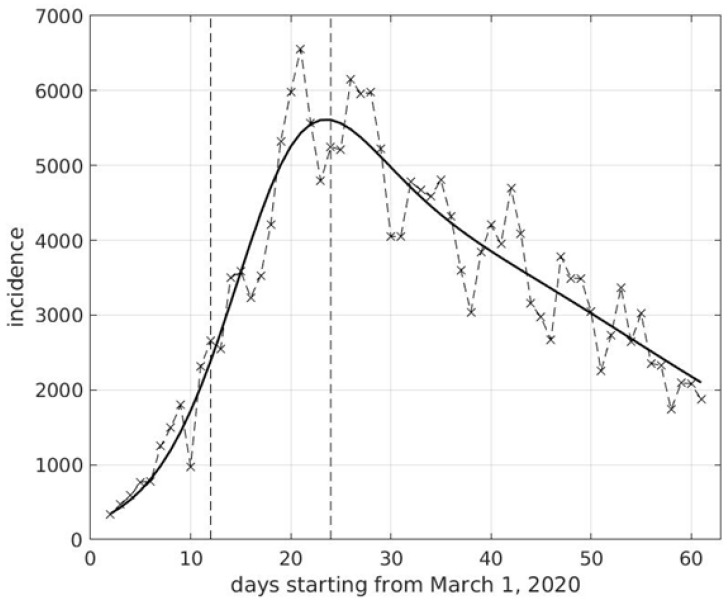
Temporal sequence of SARS-CoV-2 incidence (number of new positive cases per day) in Italy in the period March–April 2020. A fit with a generalization of the logistic model is superimposed to data. The first vertical dashed segment corresponds to March 12, the start of lockdown at national level, while the second one is the day of March 24, when the peak in the estimated incidence curve was reached.
